# Adaptation of community-based distribution of family planning services to context-specific social networks: a case of marriage counsellors in Lusaka district, Zambia

**DOI:** 10.1186/s12913-021-06422-3

**Published:** 2021-05-07

**Authors:** Lucy Nyundo, Maxine Whittaker, Lynne Eagle, David R. Low

**Affiliations:** 1 National Institute of Public Administration (NIPA), Lusaka, Zambia; 2 Public Health, Medical and Veterinary Sciences, James Cook University, Townsville, Australia; 3 College of Law Business and Governance, James Cook University, Townsville, Australia; 4 Asia Pacific College of Business and Law, Charles Darwin University, Darwin, Australia

**Keywords:** Community-based distribution, Pre-marital Counselling, Social networks, Fertility decisions

## Abstract

**Background:**

The significant contribution of community-based distribution (CBD) of family planning services and contraceptives to the uptake of contraceptives in hard-to-reach communities has resulted in the scaling-up of this approach in many Sub-Saharan countries. However, contextual factors need to be taken into consideration. For example, social network influence (e.g. spouse/partner, in-laws, and parents) on fertility decisions in many African and Asian societies is inevitable because of the social organisational structures. Hence the need to adapt CBD strategies to the social network context of a given society.

**Methods:**

Data collection involved structured interviews from August 2018 to March 2019. Randomly selected respondents (*n* = 149) were recruited from four purposively selected health facilities in Lusaka district, Zambia. Respondents were screened for age (> 15 yrs.) and marital status. A mix of categorical and qualitative data was generated. The Statistical Package for Social Sciences (SPSS®^24^) was used to carry out descriptive analysis and tests of association (Fisher’s exact) while Nvivo®^12^ was used to analyse the qualitative data using a deductive thematic approach.

**Results:**

The results indicate that pre-marriage counselling (pre-MC) influences key elements of the husband-wife relationship (*p* > 0.005), namely; sexual relationship, inter-personal communication, assignation of roles and responsibilities, leadership and authority. These elements of the husband-wife relationship also affect how spouses/partners interact when making fertility decisions. More importantly, the majority (86%) of the respondents indicated having a continuing relationship with their marriage counsellors because of the need to consult them on marital issues.

**Conclusion:**

Marriage counsellors, though hardly reported in fertility studies, are important ‘constituents’ of the social network in the Zambian society. This is because marriage counsellors are trusted sources of information about marital issues and often consulted about family planning but perceived not to have the correct information about modern contraceptives. In this context, pre-MC offers a readily available, sustainable and culturally appropriate platform for disseminating accurate information about modern contraceptives provided in a private and personal manner. Therefore, the CBD strategy in Zambia can harness marriage counsellors by recruiting and training them as community agents.

**Supplementary Information:**

The online version contains supplementary material available at 10.1186/s12913-021-06422-3.

## Background

The use of community health workers (i.e. people who are trained in family planning but have no formal clinical training) to deliver family planning services and contraceptives has been found useful in Africa, Asia and Latin America [1]. This approach, i.e. community-based distribution (CBD), overcomes the barrier of accessibility in hard-to-reach communities especially in rural areas. The key responsibilities of the community health workers are health education, distribution of contraceptives that do not require a prescription and to refer clients for clinic-based services e.g. insertion or removal of a contraceptive [1]. However, emerging evidence shows the need to adapt the CBD approach to the context [2] and to go beyond the prototype to include other factors such as improved spousal and social support, respect of client’s method choice or preference and management of side effects [3, 4]. One of the ways the CBD approach can be adapted is by understanding the architecture or composition of the social network in a given society.

In Zambia, the role and importance of marriage counsellors are well established [5, 6]. In addition to preparing couples that are about to wed and to help reconcile couples encountering marital difficulties, marriage counsellors have been identified as useful actors who can be used to empower women with safe sex negotiation skills in light of HIV risks [7]. They can also be used to disseminate information about the signs of, and screening for, cervical cancer [8]. However, studies on fertility and family planning in Zambia have not reported the actual or potential influence of marriage counsellors on couples’ fertility decisions or behaviour. Hence, in the context of fertility decisions and behaviour, the marriage counsellors in the Zambian society remain ‘hidden’ within the social network.

Therefore, this study sort to explore the influence of the Pre-MC teachings on a couple’s fertility decisions as a basis for adapting the community-based distribution of family planning services and contraceptives for the Zambian society.

## Social networks and fertility decisions

Social network theory emerged in the mid-1950s when Barnes discussed social organisation and system in a Norwegian community [9]. Today, social network theory. Which explains how attitudinal and behaviour change can be influenced by social interactions and relations among people, is used in varying research contexts to understand information transmission and diffusion of innovations [10]. This theory has been found useful in understanding fertility choices and behaviours. This is because fertility choices in part, depend upon the fertility behaviour of other persons with whom a person or couple interacts and the structure of their interaction [11].

For example, a study carried out in Poland established that social networks (e.g. family, friends and community) are more effective determinants of the diffusion of fertility behaviour than personal and demographic factors [12]. Another study in Kenya established the variance in social effects on fertility choices across different population segments [13]. Similarly, in a study carried out in Mali [14] it was established that social effects on contraceptive decisions among older and younger women differ. This Mali study established that older women (30 years and above) were prone to a wider social network influence than younger women; their social network included spouse/partner, in-laws and social associations e.g. credit schemes. This resonates with the conclusion that social influence tends to be more powerful when the group is homogeneous and the network is composed of dense and close ties [15].

Fertility decisions are prone to social effects because of social learning and influence [15]. These social mechanisms can facilitate or inhibit the uptake of modern contraceptives [11]. Social learning refers to the process by which persons obtain information to reduce uncertainty in decision making (e.g. fear of side effects and health concerns), while social influence is the control that is exerted by other persons (e.g. male spouse/partner and social disapproval of the use of modern contraceptives [13].

Although social network influence is important in fertility decisions and behaviour, research has shown that social networks mainly exert negative influence and pressure especially in African societies where male spouse/partner and social disapproval of the use of some modern contraceptive methods is prevalent [16–19]. Furthermore, young women tend to learn about both the true side effects and myths about modern contraceptives from their social network [20].

Therefore, not only does social network theory offer a basis for understanding this influence, but it also distinguishes the levels and types of influence. Social network influence is at three levels; immediate e.g. spouse/partner and relatives, community e.g. traditional and religious leaders, and national e.g. government and civil society [12]. Understanding and distinguishing between these different levels of social networks is important. This is because these levels reflect different types of support and roles. For example, the husband, friends and mother provide emotional support, while cognitive support is obtained from the mother-in-law and practical support comes from sisters-in-law and co-wives (in the case of polygamy) [21].

Consequently, network composition is crucial to the adoption and use of modern contraceptives. Although the ultimate decision-maker is the woman, she tends to engage/consult other actors (social network) along the decision-making process. The size and density of a female’s social network are important. Some women may have a dense social network which may include the male spouse/partner, in-laws, siblings, friends and co-wives (polygamous marriage) while others may have a less dense network constituted by a few people such as husband and mother [21]. The assumption is that the denser the social network the greater the influence. Of particular importance are the immediate and community levels of social network influence [12, 21].

The immediate level includes spouse/partner and close relations. The community level includes leaders, who can be categorised as formal and informal. Formal community leaders are well established in the structural arrangements of the community, for example, traditional and religious leaders, while the informal ones may not necessarily be recognised within the structural arrangements of the community but are important to a community and society, for example, marriage counsellors and mentors in society.

## Pre-marital counselling in Zambia

In Zambian society, the expectation is that ‘culturally abiding’ couples should undergo either or both religious and traditional pre-marriage counselling (pre-MC). It is the responsibility of the family (i.e. parents, guardians and close relatives) and the church to ensure that the couple complies [22]. Furthermore, the state through the Ministry of National Guidance and Religious Affairs, and the Ministry of Chiefs and Traditional Affairs supports and promotes beneficial traditional or cultural practices and norms (e.g. pre-MC) which are in line with Christian values and morals. These expectations affirm the importance of the integration of African and Christian values [23, 24] and the need for approaches, programmes and policies to be culturally sensitive [25, 26].

The advent of Christianity in Zambia towards the end of nineteenth century [27–30], among other things, led to the emergence of religious pre-MC which to date is mandatory for couples that wish to marry in church. In 1991, Zambia was declared a Christian nation and more than 85% of the Zambian population follow some form of Christian faith [30]. In response to this and the need to preserve Zambian cultural values about marriage, traditional marriage counselling has evolved by adapting its contents to the modern society and doing away with harmful marital traditional practices and teaching methods [6, 7]. Furthermore, the traditional marriage counsellors - known as ‘*Alangizi’* in one of the Zambian dialects, have established the Alangizi National Association of Zambia (ANAZ), to regulate traditional marriage counselling. However, traditional marriage counsellors are not mandated to register with ANAZ [7].

Historically, marriage counsellors were not paid anything; neither did they expect anything in return. This is because passing on indigenous knowledge from one generation to another on how to attain a successful marriage is considered to be an honour i.e. preserving cultural values and norms about marriage [6, 7]. Even though this remains important in modern Zambian society, it is now common practice to pay traditional marriage counsellors for the service or give them a token of appreciation. However, this development presents the risk of commercialisation and proliferation of unprofessional marriage counsellors [31]. The social expectation is that marriage counsellors should not overcharge, though this is subjective because there is no ceiling on the amount or fees.

Another notable trend is the proliferation of traditional marriage counsellors on social media (e.g. Facebook) which has unsettled key stakeholders including the government [32]. This is because such practices (i.e. commercialisation and sharing of pre-MC content on social media) erodes the value of Zambian culture about marital practices, norms and customs. This has prompted the Zambian government through the Ministry of National Guidance and Religious Affairs to ban such practices explaining that:

*…the trend was immoral and should never be entertained because pre and post-marital counselling is a preserve of married people or those intending to wed…. Genuine traditional counsellors don't display their teachings in public because they understand that it is culturally wrong to do so… such people do not understand the confidentially and privacy requirements of their job* [32].

Nevertheless, both traditional and religious marriage counsellors continue to play an important role in Zambian society [6, 8]. Marriage counsellors teach couples about marital issues such as proper communication, respect for family members, conflict resolution, maturity and sexual satisfaction [6]. They are socially and culturally positioned to help couples resolve marital conflicts, communication breakdown and avoid divorce due to social tendencies like male dominance and intrusion of in-laws [33]. Marriage counsellors are considered persons of good morals and character. Their characteristics include maturity, older age, success in their marriage, ability to keep secrets (confidentiality) and knowledge of cultural marital values [7]. These fundamental characteristics are used by family members in identifying a suitable counsellor for their son or daughter to prepare them for marriage.

However, studies on family planning in Zambia hardly mention or recognise marriage counsellors as key constituents of the social network. This study identifies marriage counsellors as ‘hidden actors’ within the social network. Therefore, a study to explore the potential influence of pre-marital counselling (pre-MC) teachings on fertility decisions was undertaken. This study addressed two questions;

1. How does Pre-MC influence a couple’s fertility decisions and behaviour?
2. How can Pre-MC be adapted to Zambia’s Community-based distribution system?

## Methods

Ethical requirements for this study were approved by James Cook University (No. H7242) and the University of Zambia (No. HSSREC: 2018-APRIL-015). The National Health Research Authority (NHRA) approved the additional data collection requirement because the study was on a health-related topic, namely family planning. Furthermore, the Lusaka District Health (DHO) office granted permission to collect data from the selected public health facilities.

In line with the Zambian public sector protocol and etiquette, it was important to make a courtesy call to and inform the health facility manager (i.e. the person in charge) before the commencement of the data collection within their facility. During the courtesy call, small low cost donations (e.g. vinegar used for cervical cancer screening, methylated spirit and hand washing soap) were given to the health facility. This gesture was highly appreciated- nurses facilitated data collection by assisting with the recruitment of respondents.

More than 70 % (70%) of health facilities in Zambia are state owned, followed by private (19%) and faith-based organisations and non-government organisations (2%) [34]. Majority of people access contraceptives from public facilities (81%) and private (18%) while many of the faith oriented health facilities do not offer family planning services [34, 35]. Therefore, this study comprised of three purposively selected public and one private health facility within Lusaka district, Zambia.

Apart from ownership type, these hospitals were selected to capture a range of locations, income levels and good levels of clients to assure sample sizes to be reached in the time period e.g. the high traffic at the Obstetrics and Gynaecology clinic at the private hospital (Table 1).

**Table 1:** Health facilities from which data for the study was collected, Lusaka district, Zambia 2019

Data collection point	Catchment area	No. of respondents	Type
Chilenje Hospital	Urban-Middle income	37 (excluding pilot)	Public
Bauleni Hospital	Peri-urban	37	Public
UNHCR Clinic	Peri-urban	38	Public
Medcross Hospital	Urban- High Income	38	Private

A descriptive research design and study-specific instrument (open and closed questions) was developed using Epi Info®^7^ and tested on 20 randomly selected and screened respondents. The pilot was undertaken at Chilenje hospital from August to September 2018 and the results were presented at a conference held at the University of Zambia in Lusaka [36]. A few minor adjustments such as the wording of some questions and optional responses were made. The revised instrument was used to carry out the actual study from November 2018 to March 2019.

Collecting data from both male and female respondents was important to this study because of the nature of family planning decisions among married couples (i.e. joint/interdependent). However, a higher proportion of female respondent was recruited because most of the available modern contraceptives are designed for women. Data were collected from randomly selected respondents (*n* = 149), who were screened for age (15 years and above) and marital status (engaged/married/previously married). Marital status was important because pre-MC is for persons who are about to marry (engaged), are married or previously married.

Female (80%) respondents were recruited from the cervical cancer screening points and family planning or Obstetrics and Gynaecology clinic within the health facilities. Male (30%) respondents were recruited from the waiting areas and the Outpatient Department (OPD) of the same health facilities. Written consent was obtained from the respondents and they were compensated with mobile telephone talk time/minutes worth five Zambian Kwacha (ZMW) or 0.3 United States Dollar (USD) and a refreshment e.g. water, for taking part in the study.

A mix of ordinal or categorical data (e.g. Table 3) and qualitative data (e.g. Table 4) was generated from the structured interviews which took an average of 40 min to complete. The Statistical Package for Social Sciences (SPSS®^24^) was used to carry out descriptive analysis and tests of association (Fisher’s exact). While Nvivo®^12^ was used to create the nodes denoting the themes from the qualitative data using a deductive approach. For purposes of dealing with selection bias, simple random sampling was used to recruit the male and female respondents from the purposively health facilities. Confirmation bias by the principal investigator who understands the sociocultural norms in the Zambian society was mitigated by cross checking of the data-especially the qualitative, by the three project supervisors.

The influence of pre-MC was explored using a short Likert scale. Although, the scale was short it was reliable and was carried out on an adequate sample size (*n* = 149). Exploratory factor analysis revealed two constructs: *husband-wife relationship* and *fertility decisions*. The use of both Cronbach’s Alpha and Factor analysis in testing the reliability of a scale is encouraged [37]. Therefore, the scale was subjected to a reliability test (Cronbach’s Alpha) and the results were *p* = 0.613 for the husband-wife relationship and *p* = 0.514 for fertility decisions (Table 2).

**Table 2:** Scale reliability test for the study, Lusaka district, Zambia, 2019

Construct	Cronbach’s Alpha	Mean inter-correlation	No. of Items
Husband-wife relationship	0.613	0.288	4
Fertility decisions	0.514	0.351	2

Ideally, the Cronbach’s Alpha results (*p*-value) range from 0.70 to 0.95. However, this range should be viewed with caution because of variances in the length and dimension of scales [37]. For example, some argue that there is no lower limit to the coefficient [38]. The closer Cronbach’s alpha coefficient is to 1.0 the greater the internal consistency of the items in the scale. For constructs that have less than ten [10] items, it is common to get a low Alpha (anything above 0.005 is acceptable), in such cases reporting the mean inter-item correlation is recommended [39]. In this study the mean inter-item correlation was 0.288 (husband-wife relationship) and 0.351 (fertility decisions) which both fall within the recommended range of 0.2 to 0.4.

## Results

Nearly all respondents (94%) irrespective of their demographic profile (i.e. gender, age, education, residential area, tribe and church denomination), reported having undergone some form of pre-MC (Table 3). However, some demographic factors (e.g. gender and type of marriage) were associated with the type of pre-MC undertaken.

**Table 3:** Profile of respondents who took part in the study, Lusaka district, Zambia, 2019

Factor		Gender		Total	
		*Female*	*Male*	Count	Percent
Age	15–20	4	0	4	3%
	21–29	35	10	45	30%
	30–39	39	9	48	32%
	40–49	19	11	30	20%
	50–59	12	1	13	9%
	60+	9	0	9	6%
Highest level of education attended	Never been to school	3	0	3	2%
	Primary	38	3	41	28%
	Secondary	49	16	65	44%
	Tertiary (College & University)	28	12	40	27%
Residential area	Urban High Income	14	1	15	10%
	Urban Middle Income	25	4	29	19%
	Peri-urban	54	16	70	47%
	Urban- Mixed income	25	10	35	23%
Relationship status	Engaged	6	5	11	7%
	Married (Civil, religious, customary)	90	24	114	77%
	Previously married (divorced/widowed	22	2	24	16%
Tribe	Bemba	21	6	27	18%
	Chewa	12	4	16	11%
	Kaonde	3	0	3	2%
	Lozi	4	0	4	3%
	Lunda	5	1	6	4%
	Luvale	3	2	5	3%
	Ngoni	10	2	12	8%
	Tonga	10	6	16	11%
	Other tribes	50	10	60	40%
Church denomination	Pentecost	43	8	51	34%
	Catholic	21	7	28	19%
	Seventh day Adventist	13	7	20	13%
	United Church of Zambia (UCZ)	12	3	15	10%
	Jehovah’s Witness	9	0	9	6%
	New Apostolic	6	2	8	5%
	Other (e.g. Anglican, Baptist)	14	4	18	12%
Type of pre-MC undertaken	Religious	6	4	10	7%
	Traditional	61	12	73	49%
	Both	13	10	56	38%
	Other (e.g. Commercial)	1	0	1	1%
	None	4	5	9	6%

Fisher’s Exact test found a significant association between the type of pre-MC undertaken and gender (*p* = 0.037) and type of marriage (*p* = 0.001). This relationship is plausible because for couples who wish to wed in a church (i.e. religious marriage), undertaking religious pre-MC is mandatory but they may choose not to undertake traditional marriage counselling. At the same time, undertaking traditional pre-MC is expected of every woman but not every man.

*It is mandatory especially if you want to go through the church (marriage blessing) …* (R41, Female).

*As a woman, it is traditional for me to do so* (R115, Female).

A few respondents (6%) did not undergo any form of pre-MC for a range of different reasons such as going against social standards (e.g. getting pregnant before marriage, cohabiting) or not having the resources/close relatives to organise the pre-MC;

*…my parents chased me to go to the person who got me pregnant and they refused to look for someone to teach me* (R20, Female).

*…we just met at the bar and we started living together* (R46, Male).

*I did not have people to organise someone to teach me because my parents are dead and my grandmother, the one who raised me is also dead* (R25, Female).

Among respondents who underwent any form of pre-MC, 52% reported having undertaken the traditional counselling, 7% undertook the religious type and 40% did both religious and traditional. One respondent reported an unusual type of marriage counselling i.e. “professional marriage counselling”, offered by a private firm in Lusaka.

Although undergoing pre-MC is socially mandatory and expected of every couple that wants to wed in a culturally correct procedure, pre-MC is beneficial or useful for a couple as stated by respondents:

… *it is good in the sense that it empowers you to handle problems in marriage as they occur* (R5, Female).

*Even though I regret getting married I do not regret undergoing marriage counselling because it helped me to grow and become mature* (R19, Female).

*What I was taught is what I am experiencing in my marriage* (R14, Male).

The personal benefit may explain why nearly all respondents (99%) who had undergone any form of pre-MC indicated that they would recommend it to others. Furthermore, more than half (71%) of the respondents recommended both types of pre-MC because:

*Traditional is practical like how to cook what your spouse likes and how to take care of children while the religious one teaches spiritual things like how to pray* (R125, Female).

*Traditional values are good and at the same time we are Christians so the principles of the Bible must also be appreciated* (R12, Female).

The majority (94%) of the respondents who underwent any form of pre-MC do not regret having participated. The few (4%) respondents who expressed regret gave reasons such as bias of the traditional teachings, mismatch with the modern way of life and the emerging practice of openly sharing the teachings on social platforms as explained by one respondent.

*Marriage is difficult to handle in our generation so I feel it did not help me much and the problem is that it is now public knowledge because they are now sharing on WhatsApp and Facebook* (R130, Female).

On the other hand, seven out of the nine (7/9) respondents who reported not undertaking any form of pre-MC regretted this decision, explaining that;

*There is a lot that I would have liked to learn like how to cope with my husband's character* (R139, Female).

*I regret so much because there are times when I wish I had known more* (R24, Male).

*Marriage is good but certain things are difficult. I wish I had someone to teach me and talk to. Anyway, I have come to learn a few things through church meetings* (R19, Female).

### Content of pre-MC

The thematic analysis shows that the topics covered during pre-MC; irrespective of the type (traditional or religious) are pillars of marriage, bedroom matters, home management, social conduct, social norms and family planning (Table 3). However, the level of emphasis and details of topics covered during pre-MC may vary.

*I am told that the religious teachings are not as detailed as the traditional ones and my friends have convinced me that I should do the traditional one*, (R9, Engaged, Female).

Furthermore, it was evident that traditional pre-MC places emphasis on the sexual relationship and house chores while the religious one is on religious values of marriage. A few respondents reported that family planning was covered during pre-MC. The teachings on family planning addressed the importance of birth spacing, costs associated with childbearing and contraceptive methods (Table 4). However, the majority of the respondents did not report family planning as being one of the topics covered during pre-MC.

**Table 4:** Thematic analysis on topics covered during pre-MC, Lusaka district, Zambia 2019

Theme	Verbatim examples
**Pillars of Marriage** e.g. Love and care, respect, communication, problem resolution, faithfulness and forgiveness.	*Respect for my husband know what my husband wants and likes, love faithfulness* (R138, Female). *How to take care of each other, how to solve problems…* (R64, Female). *How to respect elders, how to love my wife and how to treat in laws* (R20, Male). *How to live with a woman no fighting or chasing the woman*…(R135, Male).
**Bedroom Matters** e.g. Sexual relationship, personal grooming and confidentiality.	… *how to dance or issues of the bedroom* (R29, Female). …*how to keep secrets* (R48, Female). …*how to please my husband sexually* (R5, Female).
**Home Management** e.g. House chores, income (upkeep), budgeting and raising children	*All aspects of marriage such as managing the home, providing for the home, sex and so on* (R52, Male). …*how to cook how to care for a man because men do not know how to look after themselves* (R65, Female). *How to budget with my partner*… (R100, Male).
**Social Conduct** e.g. Behaviour and company, respect and support extended family.	…*how to receive relatives, how to respect in laws and how to live happily with other people* (R132, Female). *Once married the type of people to associate with…* (R100, Male). *How to take care of the family, how to behave like someone who is married, duty and responsibility of a husband* (R32, Male).
**Social Norms** e.g. Submission, humility, obedience, leadership and authority.	*Submission and respect for your husband*… (R131, Female). … *how to be obedient and humble*… (R21, Female). … *that the man is the head of the house* (R66, Female). …*how to kneel before him and thank him* (R91, Female).
**Family Planning** e.g. Births spacing, Family size and FP Methods	*They told me once I give birth I should go back to them they show me which family planning to use and they told me not to continuously have children* (R38, Female). …*they taught me about it and the need to ensure that we weigh ourselves and not just have children anyhow* (R63, Female). *They explained to me why it was important for us to space births so that they grow properly* (R91, Female).

Half (50%) of the respondents indicated that they would consult marriage counsellors about family planning while 38% indicated that they would not. The reasons for not engaging with marriage counsellors about family planning or reproductive health were based on the perception that they may not have the right information or knowledge about family planning.

*Marriage counsellors do not have the technical knowledge about these matters even though they know the different methods* (R67, Female).

On the other hand, respondents also indicated that they would consult them because of their personal lived experience and in the event of a conflict arising out of fertility decisions.

*I think they have experience and knowledge so I would ask them* (R79, Female).

*My husband used to oppose me using a contraceptive so at some point I had to seek the advice from the church marriage counsellors* (R132, Female).

Furthermore, marriage counsellors may still be a good source of information about traditional and natural methods because it is evident that they played a significant role in passing on indigenous knowledge about birth spacing methods.

*I did consult them because back in the days there were no family planning pills so I went back to ask what I could use* (R2, Female).

*They actually talked about this during the marriage counselling and discouraged me against using contraceptives other than safe days* (R27, Female).

### Influence of pre- MC on husband-wife relationship and fertility decisions

The frequency analysis on the data from the question ‘*On a scale of zero (strongly disagree) to five (strongly agree); pre- MC influences a couples understanding of (a) roles and responsibilities, (b) leadership and authority, (c) sexual relationship, (d) communication, (e) fertility decisions and (f) contraceptive choice* ’, shows that majority of the respondents were of the view that pre-MC influences a couple’s understanding of roles and responsibilities (81%), leadership and authority (79%). It also influences their sexual relationship (85%) and the way they communicate (83%).

More than half (54%) of the respondents reported that pre-MC does not influence the fertility decision –specifically the family size decision. This is consistent with the respondents’ view that it is not necessary to consult third parties about the family size decision but women tend to do so about their contraceptive choice (e.g. with a close friend, mother and marriage counsellors). Which explains the proportion (32%) of respondents who strongly agreed that pre-MC influences the contraceptive decision.

Given that there are two types of pre-MC (traditional and religious), the influence of the type of pre-MC on the four elements of the husband-wife relationship was explored using Fisher’s exact test. The test results (Table 5) show a statistically significant association between the type of pre-MC and the influence on the elements of the husband-wife relationship (*p* < 0.005). However, the study did not establish which type of pre-MC had greater influence, especially because many respondents reported having benefited from both types and recommended undertaking both.

**Table 5:** Fisher’s exact test results on the influence of pre-MC, Lusaka district, Zambia, 2019

Element	Type of pre -MC	Family size decision	Contraceptive decision
	*p value*	*p value*	*p value*
Sexual Relationship	0.000	0.034	**0.096**
Communication	0.001	0.028	0.001
Roles & Responsibilities	0.000	0.024	**0.254**
Leadership & Authority	0.001	0.008	**0.313**

Fisher’s exact test revealed an association between the family size decision and the elements of the husband-wife decision (Table 5) but this association was not very strong for some of the elements (0.001 > *p* < 0.005). The results also show that there was no statistically significant association (*p* > 0.005) between the contraceptive decision and three elements of the husband-wife relationship (i.e. sexual relationship [*p* = 0.096], roles and responsibilities [*p* = 0.254], leadership and authority [*p* = 0.313]). However, there was a statistically significant association with communication (*p* = 0.001).

This is plausible because, unlike the family size decision, the female spouse makes the ultimate decision on which modern contraceptive method to use, but the male spouse must first approve of and consent to the adoption and use of modern contraceptive methods. This decision making role of the male is reinforced in the counselling across all aspects of the relationship due to social norms (Table 4).

**Fig. 1:**
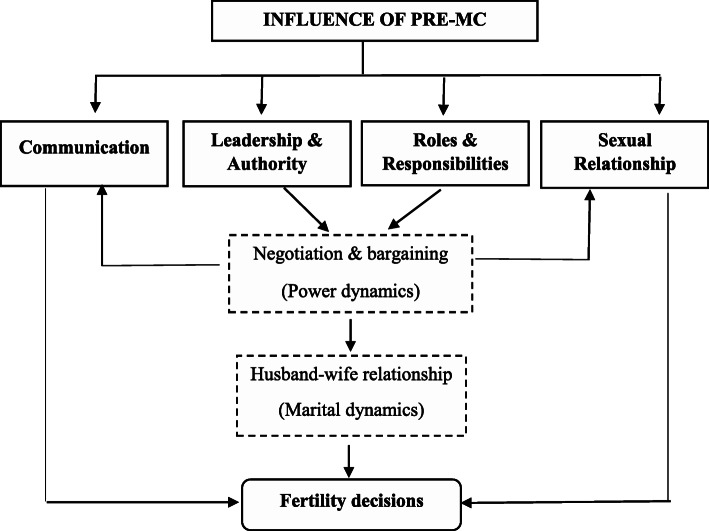
Model for the influence of pre-MC on fertility decisions based on study results, Lusaka district, Zambia, 2019. Power and marital dynamics may vary from one couple to another and from one society to another. Factors that shape the power and marital dynamics

Overall, the results indicate that pre-MC influences key elements of the husband-wife relationship, but the data does not show whether this influence is negative or positive. Nonetheless, this influence has implications because key elements of the husband-wife relationship (e.g. sexual relationship, communication, roles and responsibilities, leadership and authority) shape the spouse/partner interaction in fertility decision-making. This influence is modelled in Fig. 1.

### Structural arrangements of pre-MC

Parents and guardians are responsible for identifying and approaching the traditional marriage counsellors to counsel their daughter or son before marriage. Traditional marriage counsellors are given a token of appreciation (monetary or in-kind e.g. chicken) and paid by the parents or guardians,

*I am told it is the standard that you pay something but not something very big and it is paid by the parents* (R9, Male).

*My parents paid because they wanted me to learn and it is expected that you give the counsellors something* (R27, Female).

*Just to appreciate the knowledge and wisdom* (R32, Male)

However, some marriage counsellors do not accept payment because they perceive the passing on of cultural marital values to younger generations to be priceless and an honour as explained by one respondent:

*My marriage counsellors they saw it as an honour so they did not ask or accept the payment* (R86, Female).

This view is common among religious counsellors because they volunteer to be part of the pool of marriage counsellors at the church they attend. Therefore, the person in charge of the Church (e.g. Parish priest or pastor or catechist) allocates religious marriage counsellors to a couple upon registration of their intention to wed in church.

*The marriage counselling at church is free* (R104, Male).

In most cases, pre-MC (irrespective of the type) is held over 1 to 3 months (53%) and each session lasts 1 to 2 h (66%). More importantly, many couples continue their relationship with the marriage counsellors after they wed because of the need to consult them for other related matters as explained by some respondents;

*My marriage counsellors helped me to build my marriage and I would like to consult them when I get stuck* (R1, Female).

*I am still in touch with them because I still want to get more knowledge from them* (R5, Female).

## Discussion

Marital counselling can take a preventive or therapeutic approach [33, 40, 41]. The preventive approach is proactive – prepare the couple for realities of marriage, while the therapeutic approach is used to deal with marital conflicts as they arise. In Zambian society, the mandatory requirement of undergoing pre-MC shows the dominance of the preventative approach as reflected by the results of this study.

Pre-MC shapes the husband-wife relationship by teaching the couple the cultural or religious norms and values of Zambian marriage. This is evident from the results of this study, which show that pre-MC influences a couple’s sexual relationship and their understanding of communication, roles and responsibilities, leadership and authority.

Clearly, pre-MC is important in Zambian society irrespective of the demographic profile or parity of individuals or couples. These results affirm the conclusion that, although Zambian way of life has modernised, traditional pre-MC counselling remains valued [8]. More importantly;

*Marriage counsellors can be used to pass on information about sexual and reproductive health because they teach young women about sexual intercourse and hygiene* [8] pg. 64.

Although the results of this study are based on a case study, extant studies confirm the existence of different types of marriage counselling (i.e. Indigenous Africa, Western, Christian and Islamic) in African societies which play an important role in the survival of marriage and families [40]. Not only is the marital relationship (husband-wife) important to the success of African marriages but its key elements such as spousal communication, spouse’s/partner’s parity, perceptions and opinions about modern contraceptive and bargaining powers, are important in fertility decisions and behaviour [42–45].

Currently, some marriage counsellors include family planning as one of their topics in the pre-MC training. However, their teachings are inclined to family size and health benefits of birth spacing. In most instances, contraceptive options are not covered. The content on this particular topic (i.e. family planning) is not adequate and in some cases may be inaccurate. Nonetheless, marriage counsellors remain trusted sources of information about key marital issues.

Therefore, deliberate efforts should be made to engage and train marriage counsellors so that the content of the FP sessions during pre-MC are enriched. This can positively influence a couple’s fertility decisions and behaviour. FP interventions should include advocacy for family planning and contraceptive options to be included as a key topic in pre-MC sessions. For example, in response to the advocacy for religious leaders to help accelerate the uptake of family planning in Zambia [46], rreligious marriage counsellors can be easily incorporated into the country’s CBD system. This fits in well with and responds to the call for FP interventions in Sub-Saharan Africa to scale up and go beyond the generic CBD framework [3] by adapting to context specific factors (e.g. social network). In this case, though marriage counsellors are ‘hidden actors’, they are potential and suitable community agents that should be incorporated into Zambia’s family planning CBD strategy [47–49].

## Conclusion

This study shows that: pre-MC in Zambian society influences key elements of the marital relationship; marriage counsellors are respected and are important sources of information about marriage; many couples continue to have a relationship with their marriage counsellors, and undergoing pre-MC is culturally mandatory across all 72 ethnic groups (tribes). Although the results of this study are limited to a district, they do reflect a broad number of tribal groups, and religions (Table 3). The findings therefore have practical implications for FP programs because marriage continues to be an important institution, not only in Zambia but also in many other African societies.

Therefore, this study offers evidence to support the need to adapt the CBD strategy to a society’s social network. In this case, pre-MC offers a readily available, long term, socially and culturally suitable platform, to be strategically used to disseminate FP information and deliver personalised counselling within the community. Future research should consider establishing the relative influence of the types (traditional and religious) of pre-MC on fertility decisions to enhance the evidence for supporting the use of pre-MC as an information dissemination platform for FP programmes.

## Supplementary Information

**Additional file 1.**

**Additional file 2.**

## Data Availability

The dataset(s) supporting the conclusions of this article is (are) available on request from the authors.

## Abbreviations

ANAZ: Alangizi National Association of Zambia
CBD: Community based distribution
DHO: District Health Office
FP: Family planning
JCU: James Cook University
NHRA: National Health Research Authority
OPD: Outpatient Department
Pre-MC: Pre-marital Counselling
SPSS: Statistical Package for Social Sciences
UNHCR: United Nations High Commissioner for Refugees
UNZA: University of Zambia
USD: United States Dollar
ZMW: Zambian Kwacha
