# Supplementary figures and images for: Adaptation of community-based distribution of family planning services to context-specific social networks: a case of marriage counsellors in Lusaka district, Zambia
Source: BMC Health Serv Res. 2021 May 7;21:437. doi: 10.1186/s12913-021-06422-3 (PMC8106238; doi:10.1186/s12913-021-06422-3)

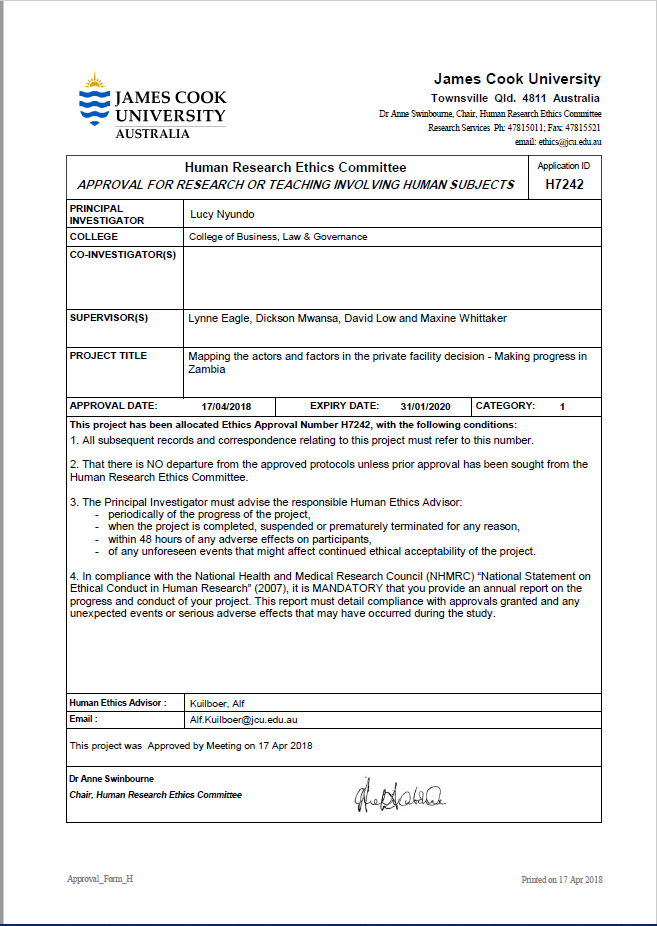


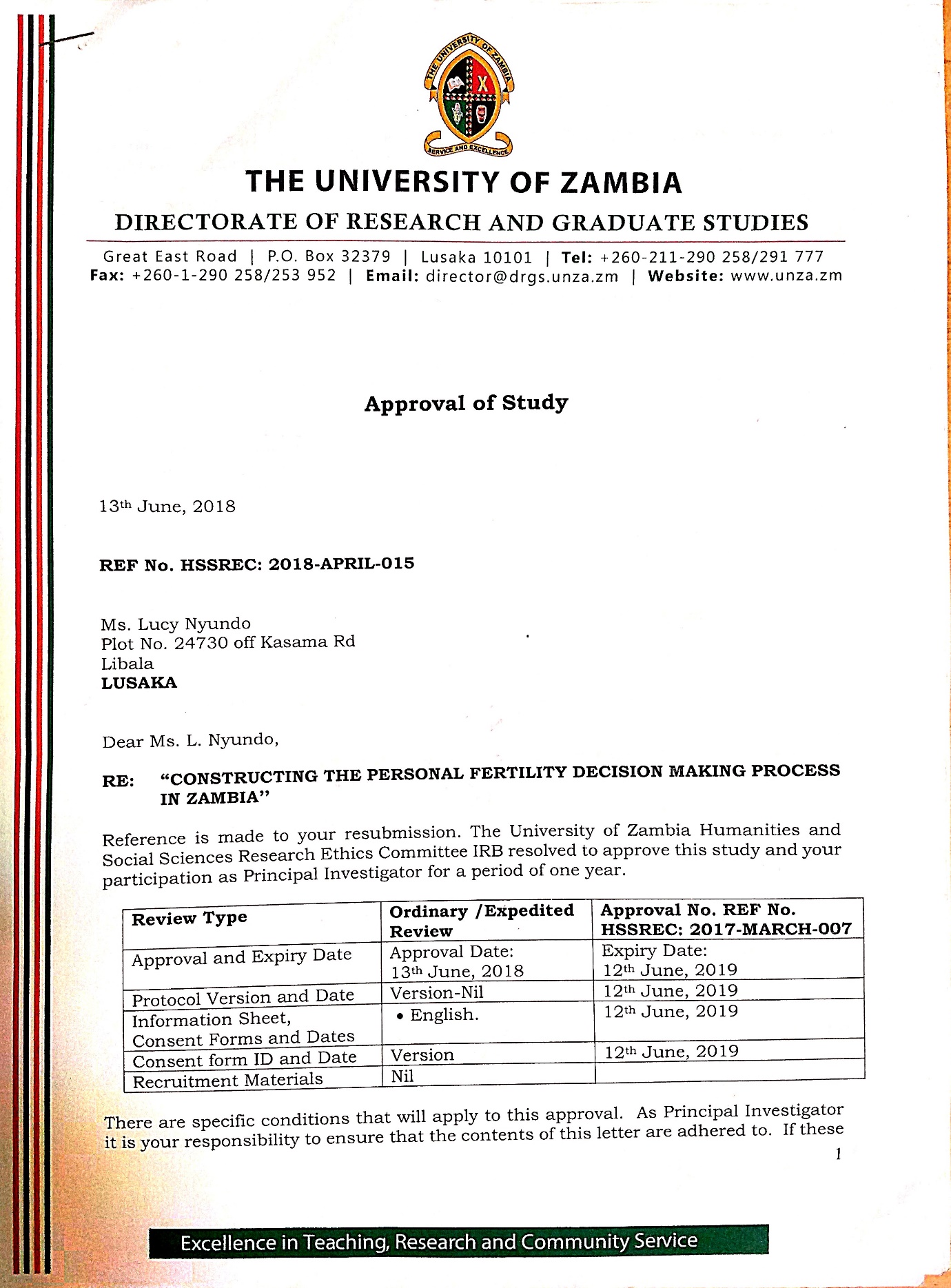


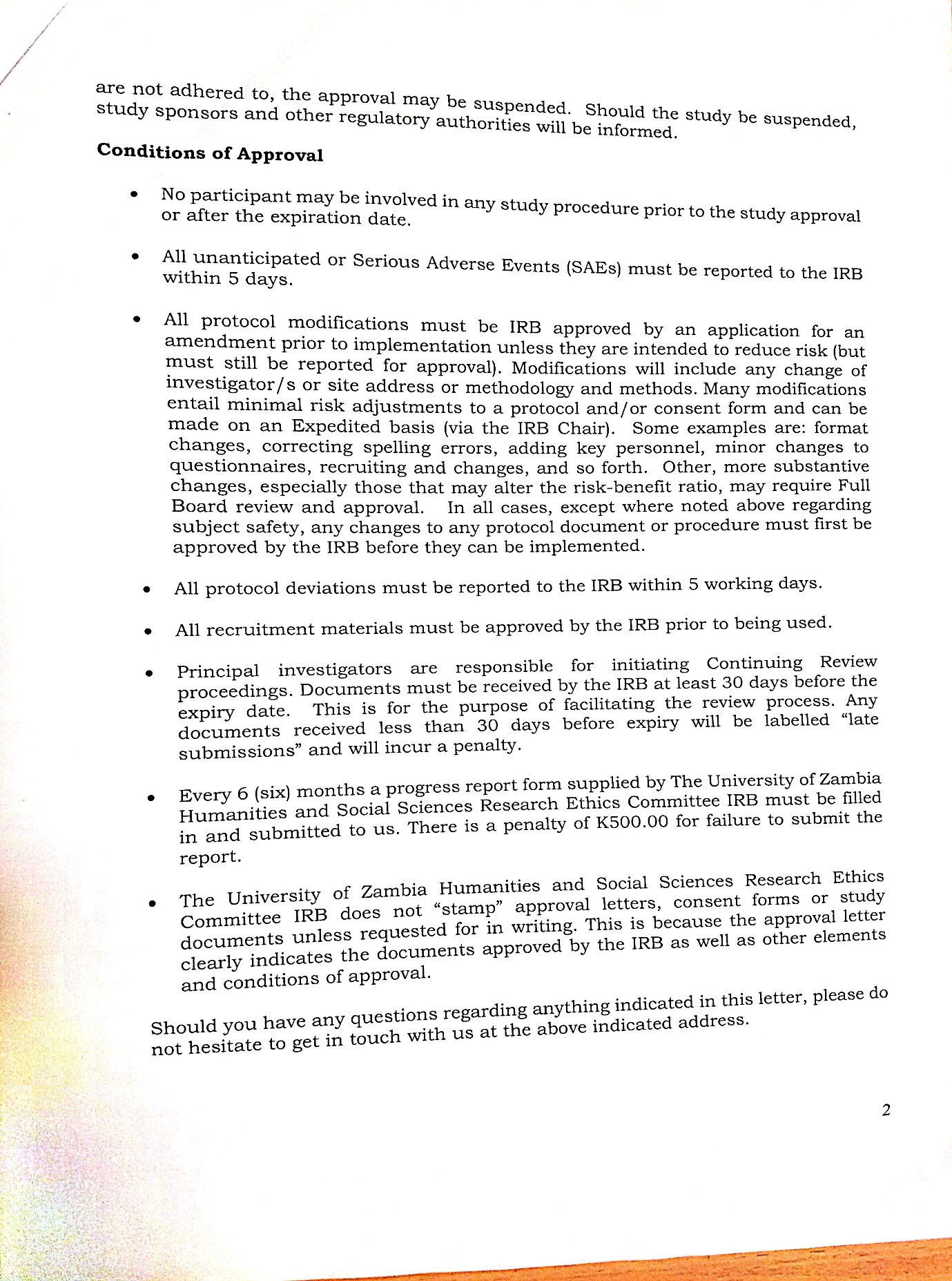


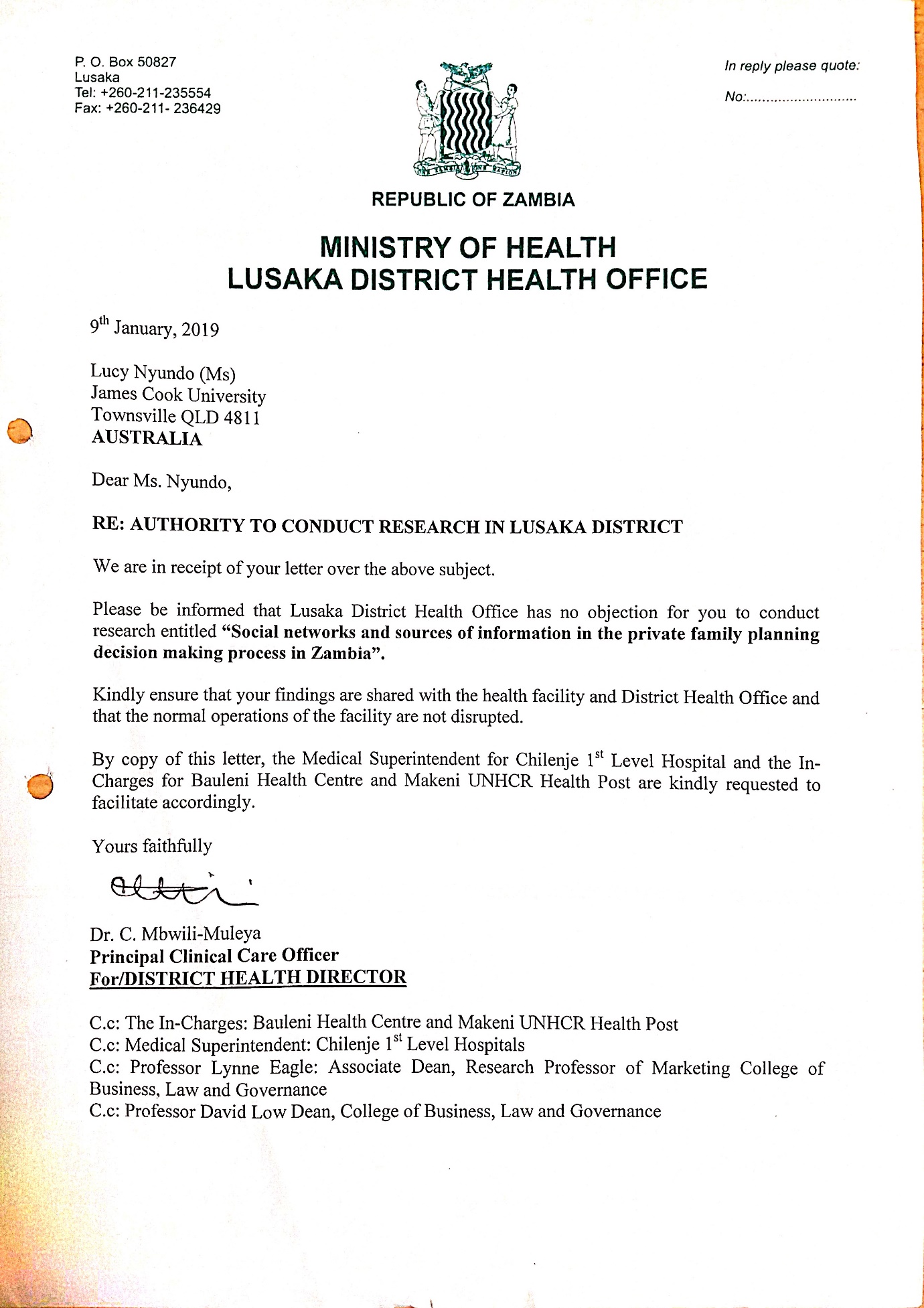

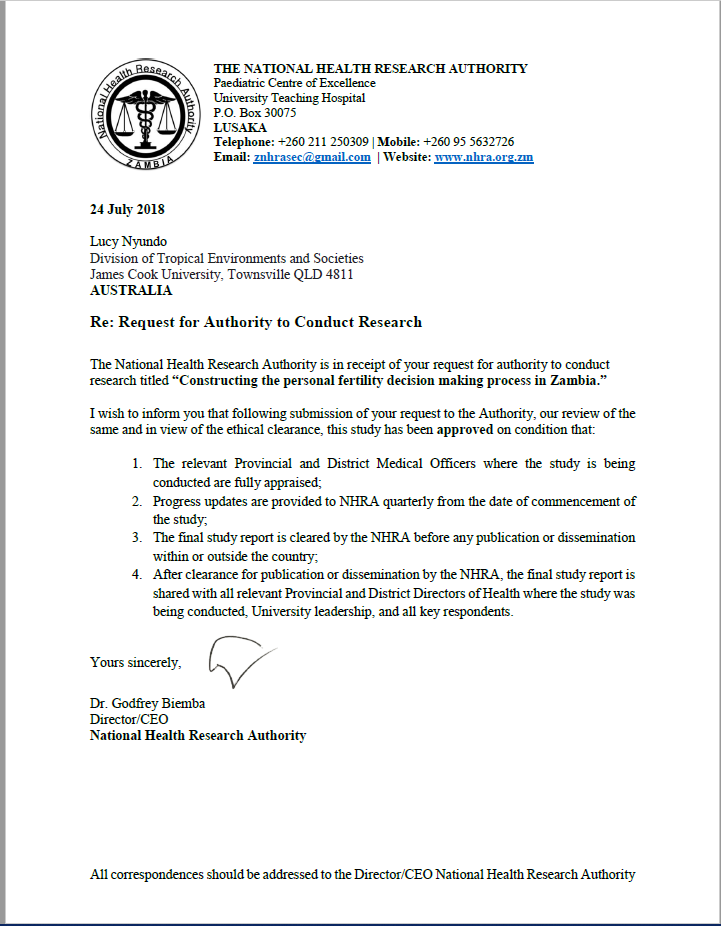

Supplement: Supplementary file 1 — Additional file 1. [file 12913_2021_6422_MOESM1_ESM.docx]
