# Supplementary material for: Adaptation of community-based distribution of family planning services to context-specific social networks: a case of marriage counsellors in Lusaka district, Zambia
Source: BMC Health Serv Res. 2021 May 7;21:437. doi: 10.1186/s12913-021-06422-3 (PMC8106238; doi:10.1186/s12913-021-06422-3)
Supplement: Supplementary file 2 — Additional file 2. [file 12913_2021_6422_MOESM2_ESM.docx]

**Questionnaire**

| SECTION | RESPONSE OPTIONS |
| --- | --- |
| **Section A: Respondent’s Profile** | |
| 1. Area of residence | Open |
| 1. Age | 15-19, 20-29, 30-39, 40-49, 50-59, 60 + |
| 1. Gender | Male, Female |
| 1. Main Household Income | Own job, Spouse/Partners job, Own business, Spouse/partners business, other, prefer not to say |
| 1. Level of Education | Never been to school, Primary, Secondary, College, University, Prefer not to say |
| 1. Religion | Christian, Hindu, Muslim, None, Prefer not to say |
| 1. Church | United church of Zambia, Pentecost, Seventh Day Adventist, Baptist, Anglican, Jehovah’s Witness, Catholic, New Apostolic, Other, Prefer not to say |
| 1. Tribe | Lozi, Bemba, Chewa, Ngoni, Tonga, Lunda, Kaonde, Luvale, Other, Prefer not to say |
| **Section B: Marriage Counselling** | |
| 1. Current Relationship Status | Civil Marriage, Customary Marriage, Religious Marriage, Polygamous Marriage, Engaged, On Separation, Divorced, Widowed, Prefer not to say |
| 1. Undergo Pre-MC | Yes, No, Prefer not to say |
| 1. Reason for undergoing Pre-MC | Expected to do so, Personal benefit, Not sure, Other (*Tick all that apply*) |
| 1. Type of Pre-MC done | Traditional, Religious, Both, Other, Prefer not to say |
| 1. Decision marker of type of Pre-MC to undertake | Myself, Church, Spouse/Partner, parents/Guardian, Close Relatives, Not sure (*Tick all that apply*) |
| 1. Cost of Pre-MC | >K200, K201-K400, K401 +, Not sure |
| 1. Person responsible for covering Pre-MC Costs | Myself, Parents/guardians, Spouse/partner, Close Relatives, Church, Not sure (*Tick all that apply*) |
|  | Why? Briefly explain: |
| 1. Duration of Pre-MC | Once/week for 6 months, Once/week for 3 months, Every day for a month, Up to counsellor, Not sure |
| 1. Duration of Pre-MC session | 1-2 hrs, 3-4 hrs, 5 hrs +, Varies, Not sure |
| 1. Pre-MC Themes/topics | Communication, Sex, Roles and Responsibilities, Family Planning, Behaviour of a married person, Budgeting(Finance), Conflict management/ Problem-solving, leadership and authority, Decision-making (*Tick all that apply)* |
|  | *Other, please specify* |
| 1. Continuing relationship with Pre-MC counsellors | Yes, No, Not sure |
|  | *Why? Briefly explain* |
| 1. Regret undergoing Pre-MC | Yes, No, Not sure, Not Applicable |
|  | Why? Briefly explain |
| 1. Recommend Pre-MC to others | Yes, No, Not sure |
|  | *Why, Briefly explain* |
| 1. Recommended type of Pre-MC | Religious, Traditional, Both, Not sure |
|  | *Why? Briefly explain* |
| 1. Reasons for not doing Pre-MC | Not in spouse/partner’s tradition or culture, no one to make the arrangements, we eloped, costly, time consuming, cohabiting, see no need, no one we/I know is doing it, not sure, prefer not to say (*Tick all that may apply)* |
|  | Other, please specify |
| 1. Regret NOT doing Pre-MC | Yes, No, Not Sure |
|  | Why? Briefly explain |
| **Section C: Influence of Pre-MC** | |
| 1. Couple’s sexual behaviour | Strongly Agree, Agree, Neither Agree or Disagree, Disagree, Strongly Disagree |
| 1. Couple communication |  |
| 1. Couple’s understanding of roles and responsibilities |  |
| 1. Couple’s understanding of authority and leadership |  |
| 1. Couple’s understanding of authority of in-laws |  |
| 1. Couple’s decision on family size |  |
| 1. Couple’s decision on contraceptive method |  |
| **Section D: Fertility Choices** | |
| 1. Current/desired fertility | 0, 1, 2, 3, 4, 5+ |
| 1. Family size decision | Ask spouse/partner, agree with spouse/partner, It is what I wanted, what my spouse/partner wants, up to God/Nature/ Not sure |
|  | *Other, briefly explain* |
| 1. Reasons for having/wanting children | Family/clan continuity, Childless life is unfulfilling, Gift from God, social expectation, Provide/care for me in old age, childless marriage is unfulfilling, love children, not sure, prefer not to say (*Tick all that apply*) |
|  | *Other reasons, please specify* |
| 1. Factors considered when making family size decision | Partner/spouse’ view, health status, income, relationship status, view of marriage counsellors, views of close relatives, age, Culture/traditional values, Religious values (*Tick all that apply*) |
|  | *Other factors, please specify* |
| 1. Average birth space between current children (months) | >12 , 13-24, 25-47, 48+, NA |
| 1. Recommended birth space | >12 , 13-24, 25-47, 48+, Not sure |
| 1. Preferred gender of a child | Boy, Girl, Does not matter, Up to God |
|  | *Briefly explain* |
| 1. FP method used | Condom, Pills, Withdraw, IUD, Implant, Emergency Pill, Herbs, Breastfeeding, Safe day, withdraw, injection, Sterilisation, being careful, not sure |
|  | *Why? Briefly explain* |
| 1. Factors considered when selecting FP method | Cost, easy to use, convenient, side effects, people ‘recommendation, other people’s’ view/experience, where to get it, religious values, health, availability, age, previous experience, privacy of use, partner/spouse views, current no. of children, relationship status, social/cultural values, effectiveness, Not sure *(Tick all that apply)* |
|  | *Other, please explain* |
| **Section E:Social Network Influence** | |
| 1. Consult spouse/partner about FP method | Yes, No, Not sure |
|  | *Why? Briefly explain* |
| 1. Consulted third parties about FP | Friend, Parents/Guardians, Church elders, marriage counsellors, health personnel, Neighbour, Sibling, In-law, Elders am close to, No one, Not sure *(Tick all that apply)* |
|  | *Other, please specify* |
| 1. Reasons for consulting third parties | Seek approval, enquire about what to do/use to prevent pregnancy, seek views about family size, ask where to get contraceptives, help resolve fertility conflict, ask about personal experience with contraceptives, seek recommendation, Not sure *(Tick all that apply)* |
|  | *Other, please specify* |
| 1. Respondents comments/suggestions about FP methods/contraceptives | Open |
